# Cerebral Surgery

**Published:** 1904-07-30

**Authors:** 


### PROGRESS IN MEDICINE AND SURGERY.

### CEREBRAL SURGERY.

(*Continued from page* 297.)

Head Injuries.—In the Hunterian lectures L. B. Rawling [6] treats exhaustively of fractures of the skull. He shows that the base perforated by numerous foramina and hollowed out to form air sinuses is the weakest part and so most liable to fracture. The lines of force applied to the vault are all conducted to the base which is frequently fractured without any fracture of the vault itself. From the data arrived at in his investigations he works out a table of the different positions and directions of forces applied to the vault and deducts therefrom the variety of basic fracture which will probably result. He finds that fractures of the base may be classified into different varieties, and that the same injury to the vault will generally produce one particular variety of basic fracture. In 215 cases of fracture of the base the mortality was 41·5 per cent. The general mortality of vault fractures is 20 to 25 per cent. Profuse and continuous hæmorrhage from the ear is in nearly every case diagnostic of an extradural hæmorrhage; in such cases there is, much more frequently than is generally supposed, hæmorrhage from the middle meningeal artery; he considers that trephining over the meningeal artery is indicated in some of these cases, and cites two instances in which the ear was plugged and bandaged for profuse hæmorrhage with resulting pressure symptoms, which were relieved by removing the plug. In his experience the escape of cerebro-spinal fluid from ear and nose is not by any means as frequent as is generally believed. Clear fluid may escape in considerable quantity without any fracture of the base, being derived from the membranous labyrinth or from the mucous membrane of the ear or nose as a result of great vaso-motor dilatation. The chemical examination will not always settle the question; cerebro-spinal fluid contains little or no albumen, but as the fluid which escapes as a result of a fracture of the skull is generally mixed with blood there will be albumen present. The fluid is nearly certainly cerebro-spinal (1) if the discharge begins within 24 hours of the accident; (2) if the discharge is colourless and profuse; and (3) if the discharge continues for several days.

The reducing substance present in cerebro-spinal fluid is only to be found at first, for if the discharge be profuse, it soon becomes merely a serous exudate. The discharge of cerebro-spinal fluid does not materially influence the prognosis. Of nerve injuries, involvement of the 7th and 8th nerves is most common; the 3rd, 4th, and 6th are sometimes, the 1st, 2nd, 3rd, 9th, 10th, 11th, and 12th are very rarely if ever injured. He describes two varieties of injury to the facial and auditory nerves: in (1) there is early and partial paralysis of the 7th with a variable degree of deafness; in (2) early and complete facial paralysis and complete deafness. In (1) the fracture passes in front of the facial nerve, which escapes injury except at the genu, where it may be temporarily compressed by clot, the membrana tympani is frequently torn, and the ossicles may be dislocated. In (2) the fracture generally results from a blow on the occipital region, the line of fracture traversing the cerebellar fossa to the outer margin of the jugular foramen, thence it cuts across the petrous bone external to the internal auditory meatus; in this course it divides the auditory apparatus, and the facial nerve is completely divided in the region of the genu. A photograph of such a fracture is reproduced, showing the open ends of the divided semicircular canals. The paralysis and deafness are permanent, and the lesion is not accompanied by hæmorrhage from the ear. The temperature chart is one of the surest guides in giving a prognosis. All cases may be divided into three groups:—In (1) there is a steady and progressive rise of temperature, in this group the prognosis is most unfavourable. In (2) at first the temperature is subnormal, then it rises to 101° or 102°, remains steady for a short time, and then it either (*a*) commences to fall, in which case the prognosis is good, or (*b*) to rise, the prognosis is then bad. In (3) the temperature is subnormal and remains so, this signifies that the patient has never recovered from the initial shock. If it is at first subnormal and then rises to normal without further elevation the injury has been but slight. He suggests trephining the cerebellar fossa in cases of basic fracture with compression symptoms. In such cases the autopsy always reveals excess of fluid in the posterior fossa, and although the cases are practically hopeless, he thinks that trephining and drainage might give a chance of recovery. He pleads for a longer treatment of head injuries by rest, thereby diminishing the chances of bad after effects. No person should return to work for at least six months. Crisp English [7] in the Hunterian Lectures treats of the after effects of head injuries. The minor ills, which mean so much to the patient, are but little spoken of in text-books, while the subject of traumatic epilepsy and paralysis receives much attention. He has followed out the history of 300 cases with a view to ascertaining how often and to what extent the patients suffer from serious sequelæ, impairment of mental powers, alteration of individuality, and what the influence is on wage-earning capacity. He protests against the common practice of describing the majority of cases in which there is loss of consciousness but no apparent fracture, as cases of concussion. It is clear, he says, that in many such cases there is a varying degree of contusion or laceration of the brain. The cases are divided into three series. Series 1 includes 100 consecutive cases of fracture, examined one year or more afterwards. Series 2, 100 consecutive cases of concussion, contusion, and laceration without evidence of fracture, also examined at a not shorter interval than one year. Series 3, miscellaneous cases, in which there was short or no unconsciousness, and the immediate effects very slight. Such cases frequently received little or no treatment and were not admitted to hospital. In estimating what proportion suffer from remote after effects the patients are divided into three classes, and the following table shows the percentage of effects in the first and second series:—

| | First Series. | Second Series. |
|---|---|---|
| Class 1. No effects ... ... | 31 ... | 48 |
| Class 2. Slight effects ... ... | 50 ... | 42 |
| Class 3. Marked effects ... | 19 ... | 10 |

His experience shows that in over 10 per cent. of cases, some degree of mental impairment, though rarely sufficient to be included under the heading of traumatic insanity, occurs. In the first series, 41 per cent., and in the second series, 20 per cent. of cases, showed a diminution in wage-earning capacity. The most frequent after effect is headache. The neuralgic form is rare and may be due to involvement of a nerve in the scar, such cases may be cured by excision of the scar. Headache, with local tenderness, is sometimes due to sclerosis of bone, and all other measures failing, trephining will often give relief. Headache which is fixed in one place without any local tenderness, is often due to meningeal adhesion, and may be relieved by operation. Mental irritability is common, insanity rare. Other symptoms most often observed are vertigo, change of disposition, disturbed sleep, increased susceptibility to alcohol and high temperatures. In nearly all cases with severe symptoms, he believes that there is organic injury to the brain, a view which is supported by autopsies on cases that have died from other causes at considerable periods afterwards; in all but the slightest cases, marks of the injury remain, the commonest being adhesions of the meninges to the bone, localised thickening of the meninges and discoloration of the cortex from old extravasated blood. Treatment by prolonged rest is very essential, otherwise incomplete recovery almost invariably results. In more than half the cases, rest and medical treatment suffice. Mercurial inunction often produces good results. A small number can be cured by operation when osteosclerosis or meningeal adhesion is the cause of the symptoms. The history of the operative treatment of traumatic epilepsy is one long tale of disappointment, and we are driven to the conclusion that we cannot cure this disease. Relief may in some cases be obtained for a time, but the fits almost inevitably return; however, in properly selected cases operation may be advised as providing a year or so of freedom. In one of Horsley's cases the patient was free for 14 years. In selecting cases only the Jacksonian type is suitable, and then only when there is definite history of injury, when the disease has not lasted more than two years, and when there is no neurotic family history. When convulsions become generalised, operation is no good. The question of traumatic insanity is fully discussed, and he concludes that, whilst some degree of mental impairment is comparatively common after injuries to the head, the changes are seldom sufficiently marked to be included under the heading of insanity. Only a small proportion of the cases of traumatic insanity are open to relief by operation, for a localising indication in an accessible region must be present. G. A. Wright [8] has published notes of 13 cases of neuralgia treated by injection of osmic acid into the nerve. The acid causes degeneration of the axis cylinders and white substance. He uses a $1\frac{1}{2}$ or 2 per cent. solution, injecting both towards the periphery and towards the centre. His cases are too recent to judge of the ultimate issue; but the immediate results as regards both harmlessness and at least temporary relief justify the use of this method before resorting to such extreme measures as removal of the gasserian ganglion. In this series nine cases were improved, two cured, and two failed. Cuthbert Wallace [9] records four cases of tetanus treated by injection of serum into the spinal theca. The results are good. All were severe cases, the incubation being eleven, eight, and six days. From one to three injections of 10 c.c. each were given. Three of the cases recovered, which is a high percentage for severe cases with short incubation periods.

[6] Lancet April 9, 16, 23. [7] Ibid., Feb. 20, Feb. 27, March 5. [8] Med. Chron., February. [9] Lancet, March 5.

